# Prickly Ash Seeds improve immunity of Hu sheep by changing the diversity and structure of gut microbiota

**DOI:** 10.3389/fmicb.2023.1273714

**Published:** 2023-10-31

**Authors:** Dengpan Li, Hai Yang, Qiao Li, Keyan Ma, Huihui Wang, Chunhui Wang, Taotao Li, Youji Ma

**Affiliations:** ^1^College of Animal Science and Technology, Gansu Agricultural University, Lanzhou, China; ^2^Gansu Key Laboratory of Animal Generational Physiology and Reproductive Regulation, Lanzhou, China

**Keywords:** Prickly Ash Seeds, 16S rRNA, gut microbiota, sheep, immunity

## Abstract

Prickly Ash Seeds (PAS), as a traditional Chinese medicinal herb, have pharmacological effects such as anti-asthma, anti-thrombotic, and anti-bacterial, but their impact on gut microbiota is still unclear. This study used a full-length 16 s rRNA gene sequencing technique to determine the effect of adding PAS to the diet on the structure and distribution of gut microbiota in Hu sheep. All lambs were randomly divided into two groups, the CK group was fed with a basal ration, and the LZS group was given a basal diet with 3% of PAS added to the ration. The levels of inflammatory factors (IL-10, IL-1β, and TNF-α) in intestinal tissues were measured by enzyme-linked immunosorbent assay (ELISA) for Hu sheep in the CK and LZS group. The results indicate that PAS can increase the diversity and richness of gut microbiota, and can affect the community composition of gut microbiota. LEfSe analysis revealed that *Verrucomicrobiota*, *Kiritimatiella*, *WCHB 41*, and *uncultured_rumen_bacterium* were significantly enriched in the LZS group. KEGG pathway analysis found that LZS was significantly higher than the CK group in the Excretory system, Folding, sorting and degradation, and Immune system pathways (*p* < 0.05). The results of ELISA assay showed that the level of IL-10 was significantly higher in the LZS group than in the CK group (*p* < 0.05), and the levels of TNF-α and IL-1β were significantly higher in the CK group than in the LZS group (*p* < 0.05). LEfSe analysis revealed that the dominant flora in the large intestine segment changed from *Bacteroidota* and *Gammaproteobacteria* to *Akkermansiaceae* and *Verrucomicrobiae* after PAS addition to Hu sheep lambs; the dominant flora in the small intestine segment changed from *Lactobacillales* and *Aeriscardovia* to *Kiritimatiellae* and *WCHB1 41*. In conclusion, the addition of PAS to sheep diets can increase the number and types of beneficial bacteria in the intestinal tract, improve lamb immunity, and reduce intestinal inflammation. It provides new insights into healthy sheep production.

## 1. Introduction

A large number of microorganisms live in the intestinal tract of sheep and are closely involved in host metabolism, immunity, tissue homeostasis, and intracellular homeostasis (Su et al., 2022). The composition and diversity of microorganisms are influenced by diet composition, host genetics, and external environment. For example, parasitic infections increase pathogenic bacteria and decrease beneficial bacteria in sheep (Chatanga et al., 2021; Liu and Yin, 2021). Studies have shown that the addition of oregano essential oil to diets increases the abundance of *Rumenococci*, *Bifidobacteria*, and *Enterococci* (Jia et al., 2022). Rumen degradable starch as a ration alters intestinal bacterial communities and carbohydrate digestibility in dairy goats (Han et al., 2021). In addition, the microbial communities in different intestinal segments of sheep have unique characteristics. According to the similarity of community composition determined by principal coordinate analysis, the microbial community of the entire intestine can be divided into two groups: small intestine and large intestine (Wang et al., 2017). Dominant microbiota from the foregut to hindgut of Aohan wool sheep has changed from Proteobacteria to Bacteroidota (Ma et al., 2022).

PAS is the seed left behind by the ripening, drying, and filtration of Chinese prickly ash. Which are rich in α- Linolenic acid plays an important role in anti-asthma and anti-thrombosis (Tang et al., 2014; Yang et al., 2014). In addition, the anti-inflammatory ability of prickly ash extract has been proven multiple times (Hou et al., 2021). An antimicrobial peptide named NP-6 in PAS inhibited all tested strains (Hou et al., 2019). PAS may inhibit lung inflammation and tissue damage during asthma (Wang et al., 2016). The research shows that Prickly Ash oil extracted from PAS has inhibited osteoclastogenesis and anticancer effects against malignant melanoma (Pang et al., 2019; Zhang B. et al., 2023). The addition of PAS to the diet was effective in improving the fatty acid composition of the longest muscle of the pig’s back (Song et al., 2020). Different flora produced by Prickly Ash oil fed to small-tailed frigid sheep affected on nutrient digestion and absorption and gut health of the host and significantly altered the abundance of rumen microbiota (Zhang et al., 2022a,b). Lamb diarrhea is a common disease caused by pathogenic microbial infection and digestive system dysfunction, which poses a significant threat to the sheep industry. Diarrhea in sheep is mainly caused by rotavirus, *Escherichia coli*, streptococcus, salmonella, clostridium welchii, and Parasitic disease. And PAS has potential anti-inflammatory, antibacterial, and antioxidant properties (Fortuoso et al., 2019; Zhong et al., 2022).

The inflammatory response promotes tissue repair and an immune response, but when the immune response is excessive or persistent, it can trigger a range of serious diseases (Neurath, 2014). Inflammatory factor refers to a class of signaling factors produced by cells in the process of inflammatory response, which can stimulate immune cells to further release other inflammatory factors (Kong et al., 2021). Among inflammatory cytokines, IL-1β is considered a typical multifunctional cytokine that affects virtually all types of cells, whether acting alone or in combination with other cytokines. IL-1β is essential for cellular defense and tissue repair in almost all tissues, and has been implicated in pain, inflammation, and autoimmunity (Zhou et al., 2019). IL-1β is also involved in neuroprotection, tissue remodeling and repair (Tran et al., 2023). TNF-α enhances macrophage activity and killing function, and enhances the ability of macrophages to promote immune response; it also has a promoting effect on the activity and aggregation of neutrophils localized in inflammation (Zhang et al., 2021). IL-10 is also an important immunomodulator, which mainly inhibits and terminates inflammatory immune responses by inhibiting monocyte and macrophage activation (Bouquet et al., 2020).

In this study, a total of 60 samples from the intestinal tract (duodenum, jejunum, ileum, cecum, and colon) of normally fed and PAS-intervened sheep were studied for a comparative study of bacterial community structure and distribution by using 16 s rRNA full-length sequencing. Relevant inflammatory factors were then validated using ELISA kits. The aim was to complement the incomplete study of PAS on the intestinal flora of sheep and to provide a theoretical basis for the application of PAS to the regulation of intestinal health in ruminants.

## 2. Materials and methods

### 2.1. Ethics statement

All experimental designs and procedures were approved by the Animal Care Committee of Gansu Agricultural University (GSAU-AEW-2020-0057). in accordance with the Guidelines for Animal Care and Experimental Procedures established by the Ministry of Science and Technology of the People’s Republic of China (Approval No. 2006-398).

### 2.2. Animals and sample collection

During the study, 12 weaned male lambs (aged 90 ± 5 days; 25.66 ± 3.03 kg body weight) of Hu sheep were selected from Dongxiang County Dawan Breeding Farmers’ Specialized Co-operative Society (Linxia City, Gansu Province, China). The nutrient composition of PAS is shown in Table 1. The composition and nutrient levels of the basal rations of the CK and LZS groups are shown in Table 2. All lambs were fed according to standard livestock management practices. During the pretest period of 10 d and the LZS period of 90 d, the lambs were fed the total mixed ration (TMR) at 07:00 and 18:00 every day, and during the feeding period, the lambs were fed and watered freely, and the pens were disinfected regularly. All lambs were euthanized early in the morning of day 91. Fresh intestinal contents (~10 g) and intestinal tissue samples (~15 g) were collected from the duodenum, jejunum, ileum, cecum, and colon. In order to distinguish different intestinal segments, duodenal specimens from the CK and LZS groups were recorded as DuCK1-DuCK6 and DuLZS1-DuLZS6, respectively; jejunal specimens were recorded as JeCK1-JeCK6 and JeLZS1-JeLZS6, respectively; ileal specimens were recorded as I1CK1-I1CK6 and I1LZS1-I1LZS6, respectively; cecum specimens were recorded as CeCK1-CeCK6 and CeLZS1-CeLZS6, respectively; colon specimens were recorded as CoCK1-CoCK6 and CoLZS1 and CoLZS6, respectively. Samples were preserved in 50 mL sterile, enzyme-free centrifuge tubes and immediately placed in liquid nitrogen, then transferred to a −80°C ultra-low-temperature refrigerator for storage.

**Table 1:** Content of conventional nutrients in Prickly Ash Seeds (dry matter basis).

Items	Content/%	Items	Content/%
Crude fat	22.50	ADF	26.35
Crude protein	15.60	Ca	0.26
NDF	40.23	P	0.47
DM (air-dry basis)	90.60		

**Table 2:** Composition and nutrient levels of basal diet (dry matter basis).

Diet ingredient	CK	LZS
**Ingredients DM %**		
Corn	28	28
Wheat bran	12	12
Soybean meal	14	14
Rapeseed meal	4	4
Prickly Ash Seeds	0	3
Alfalfa hay	13	10
Wheat straw hood	10.3	10.3
Whole corn silage	14	14
Premix	1	1
Bicarb	1	1
NaCl	1	1
Limestone	1	1
CaHCO3	0.7	0.7
Total	100	100
**Nutrient levels**		
Metabolic energy (MJ/kg)	9.75	9.76
Crude protein (%)	15.38	15.35
Crude fat (%)	2.50	3.13
NDF (%)	28.35	28.22
ADF (%)	16.27	16.20
Ca (%)	0.97	0.93
P (%)	0.52	0.53

DM, dry matter; NDF, neutral detergent fiber; ADF, acid detergent fiber.

The premix provided the following per kg of diets: Fe 75 mg, Cu 14 mg, Zn 80 mg, Se 0.25 mg, I 0.25 mg, Co 0.3 mg, Mn 400 mg, VA 3000 IU, VD 500 IU, VE 200 IU.

ME was a calculated value, while the others were measured values.

### 2.3. Full-length 16S rRNA sequencing

Genomic DNA was extracted from the contents of duodenum, jejunum, ileum, cecum and colon using the TGuide S96 Magnetic Bead Method Soil and Fecal Genomic DNA Extraction Kit (Tiangen Biotechnology (Beijing, China) Co., Ltd.) according to the instruction manual, and the concentration of the DNA was determined by Nanodrop, and the purity of the DNA was determined by agarose gel electrophoresis, which was qualified and used for the amplification of full-length 16S rRNA gene amplification.

After extracting the total DNA of the samples, specific primers with Barcode were synthesized according to the full-length primer sequences (27F: AGRGTTTGATYNTGGCTCAG; 1492R: TASGGHTACCTTGTTASGACTT), and the products were purified, quantified, and normalized to form a sequencing library (SMRT Bell). The constructed libraries were first subjected to library quality control, and the qualified libraries were sequenced by PacBio Sequel II. Sequencing was performed by Biomarker Technologies (Beijing, China).

### 2.4. Enzyme-linked immunosorbent assay

Corresponding intestinal tissues (18 samples) from the jejunum, ileum, and cecum were selected for enzyme-linked immunosorbent assay. Three inflammatory factors were measured by double-antibody sandwich ELISA provided by Jingmei Biotechnology Co., Ltd. (Jiangsu, China): TNF-α (JM-07817S1), IL-1β (JM-00413S1), and IL-10 (JM-07814S1).

### 2.5. Bioinformatics analysis and statistics

After exporting the CCS (Circular Consensus Sequencing) file through smrtlink analysis software. Firstly, the Raw-CCS sequence data were obtained based on Barcode sequence identification, followed by CCS filtering using cutadapt (version 2.7) software (Bolger et al., 2014), and finally, the chimeric sequences were identified and removed to obtain Effective-CCS sequences using UCHIME (v8.1) software. The Reads were clustered using Usearch software (v10.0) (Edgar, 2013) at 97.0% similarity level, divided into OTUs/ASVs (hereafter referred to uniformly as Features). Use Venn plots to show the number of shared, unique features between individual bowel segments or groups. Using Silva.138 as a reference database, we annotated the feature sequences taxonomically using a simple Bayesian classifier to obtain the taxonomic information of the species corresponding to each feature, and then counted the composition of each sample community at the phylum level. QIIME software was used to generate species abundance tables at different taxonomic levels, which were then plotted as community structure maps at each taxonomic level of the samples using R language tools. Using QIIME2 software, the Alpha diversity index of the samples was evaluated and dilution curves were plotted (Wang et al., 2012); Beta diversity analysis was used to compare the magnitude of differences in species diversity (community composition and structure) among samples. Sample hierarchical clustering (UPGMA) trees, sample clustering heatmaps, and sample PCoA plots under the corresponding distances are obtained based on the distance matrix; Statistically different Biomarkers were searched for between groups by intergroup sample LEfSe analysis, to further measure differences in species abundance composition between samples (groups); Finally, the 16S rRNA gene sequences were analyzed for functional prediction in the KEGG database using PICRUSt2.

Correlation analysis was performed using Spearman correlation coefficient (Spearman correlation); *r* < 0 means negative correlation, *r* > 0 means positive correlation, *p* > 0.05 means insignificant difference, *p < 0.05 means significant difference, **p < 0.01 means the difference is highly significant.

## 3. Results

### 3.1. Sequencing results and bacterial diversity of the whole gut

**Figure 1:**
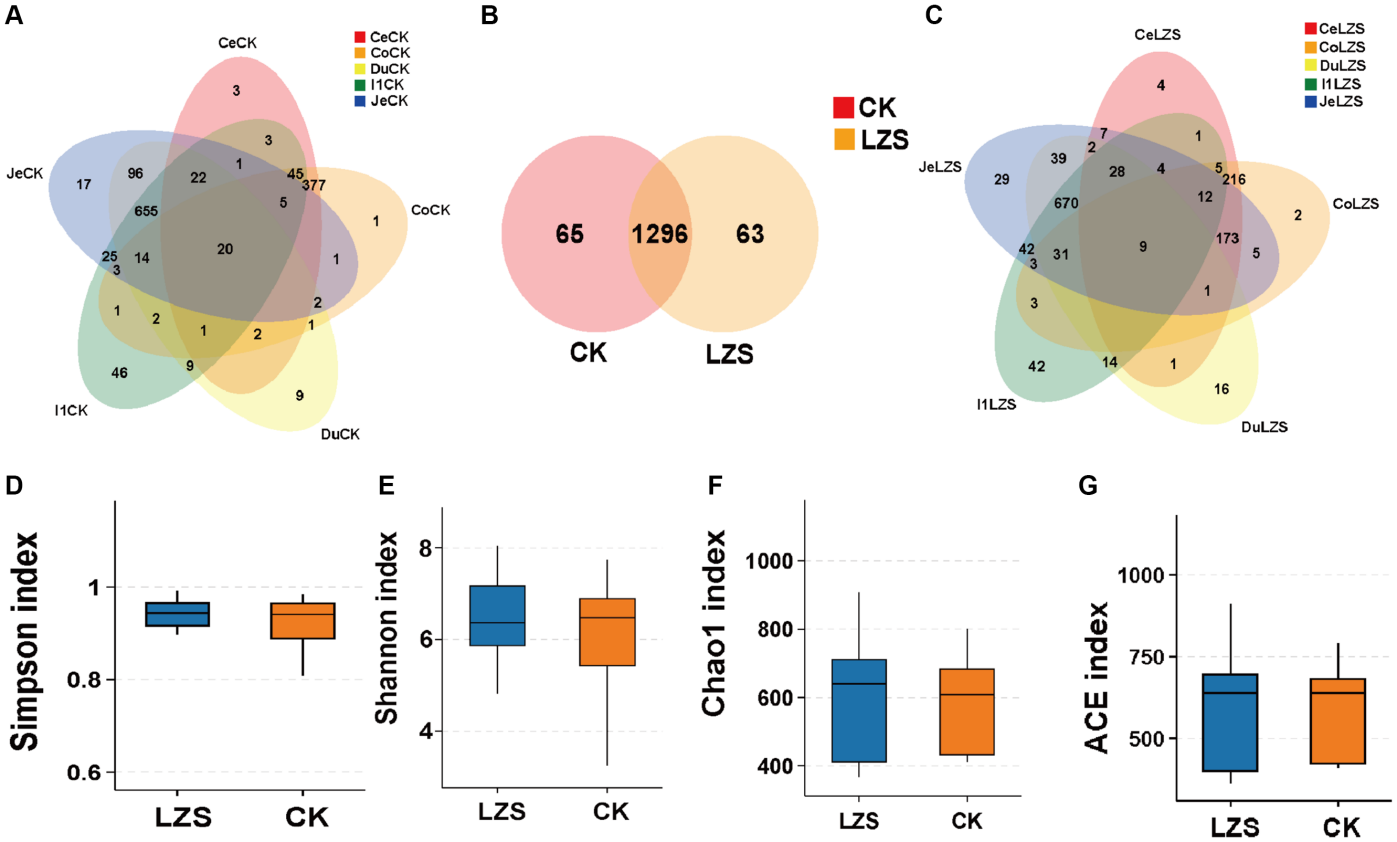
Sequencing results and statistical analysis of diversity. **(A)** Venn diagram showing the OTUs shared by each intestinal segment in the CK group. **(B)** Venn diagram showing OTUs shared in the gut between the CK and LZS groups. **(C)** Venn diagram showing the OTUs shared by each intestinal segment in the LZS group. **(D–G)** The simpson, shannon, Chao1, and ACE indices for the six bowel segments. Adjusted *p < 0.05 and **p < 0.01 by Student’s T test.

We collected five intestinal segment sites (duodenum, jejunum, ileum, colon, and cecum) and obtained a total of 60 samples for sequencing. A total of 1,200,134 CCS sequences were obtained by Barcode identification, with at least 11,700 CCS sequences per sample and an average of 20,002 CCS sequences. First, we analyzed the number of features in the CK and LZS groups. The total number of intestinal features in the CK and LZS groups was 1,424, the number of shared OTUs was 1,296, and the number of observed features in the intestines of the CK and LZS groups was 1,361 and 1,359, respectively, with no significant difference (Figure 1B). Rarefaction curves flatten out and all sequencing data are reasonable (Supplementary Figure S1). Next, we analyzed the number of features in different intestinal segments for the CK and LZS groups separately. Except for the ileum, the number of features unique to other intestinal segments was more in the LZS group than in the CK group. And the number of features contained in each intestinal segment in both CK and LZS groups was jejunum > ileum > duodenum > cecum > colon (Figures 1A,C).

To prove the accuracy of the analyzed results, we performed intestinal microbial diversity analysis. The results showed that the addition of PAS to the diet was able to influence gut microbial richness and diversity. The Shannon, Simpson, and Chao1 indices of the LZS group were higher than those of the CK group (Figures 1D–F), indicating that feeding PAS increased the diversity and abundance of gut microorganisms. However, the ACE index was lower in the LZS group than in the CK group (Figure 1G). The dilution curves of both the LZS group and the CK group flattened out as the number of sequencing strips increased. The results showed that the sequencing data were sufficient to cover all bacterial communities.

### 3.2. Cluster analysis and microbial composition of the intestine

**Figure 2:**
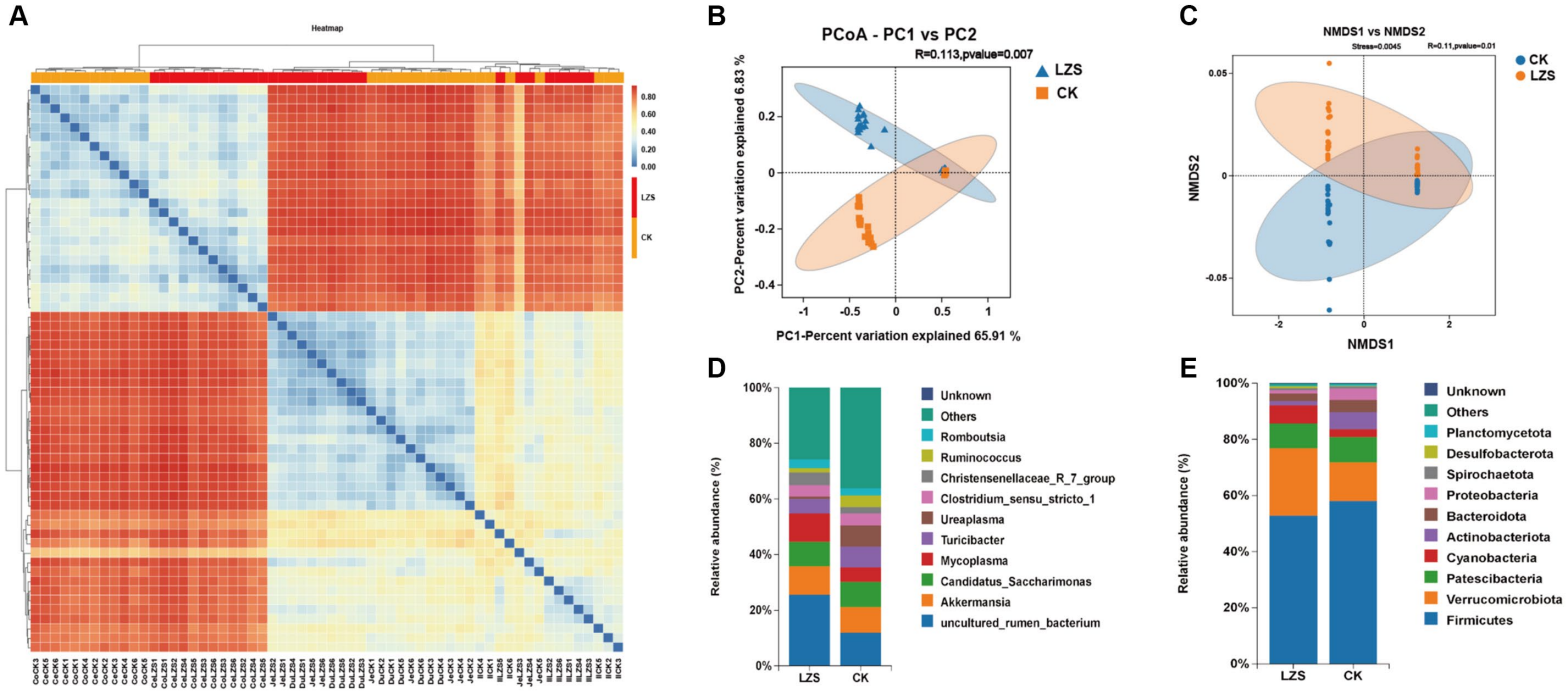
Cluster analysis. **(A)** Heatmap of species abundance at genus level. **(B)** Principal coordinate analysis (PCoA) based on all samples. **(C)** Non-MetricMulti-Dimensional Scaling (NMDS) based on all samples. **(D)** The genus-level microbial composition of the intestinal segments of the CK and LZS groups. **(E)** The phylum-level microbial composition of the intestinal segments of the CK and LZS groups.

PCoA and NMDS analyses revealed a significant separation of the microbial population structure in the gut of the LZS group from that of the CK group (Figures 2B,C). Next, we performed a heat map analysis of clustering at the genus level, which showed that the cecum and colon clustered into one group, while the duodenum, jejunum, and ileum clustered into one group (Figure 2A). Additional species distribution analysis of all samples at the genus level revealed that the large and small intestinal segments of sheep lambs were differentially affected by PAS (Supplementary Figure S2).

In the analysis of microbial composition, we focused on exploring the top 10 bacteria at the phylum and genus level. The level of microorganisms at each taxonomic level was analyzed. At the phylum level (Figure 2E), Firmicutes were both dominant bacterial gates in the CK group (58.10%) and the LZS group (52.82%). There were differences in the proportions of Verrucomicrobiota (24.06% in the LZS group and 13.78% in the CK group), Cyanobacteria (6.45% in the LZS group and 2.71% in the CK group), and Desulfobacterota (0.75% in the LZS group and 0.18% in the CK group) in the CK and LZS groups. This suggests that these bacteria are more active in the LZS group. At the genus level (Figure 2D), the dominant genera in the CK group were *uncultured_rumen_bacterium* (11.95%) and *Akkermansia* (9.26%), and the dominant genera in the LZS group were *uncultured_rumen_bacterium* (25.67%) and *Mycoplasma* (10.18%). In addition to this, most of the annotated bacteria were more abundant in the LZS group compared to the CK group. For example, *Mycoplasma* (10.18% in LZS group, 5.19% in CK group), *Christensenellaceae_R_7_group* (4.55% in LZS group, 2.25% in CK group), *Clostridium_sensu_stricto_1* (4.09% in LZS group, 4.23% in CK group) and *Romboutsia* (3.01% in LZS group, 2.57% in CK group).

### 3.3. Microbial communities and metabolic pathways in the gut

**Figure 3:**
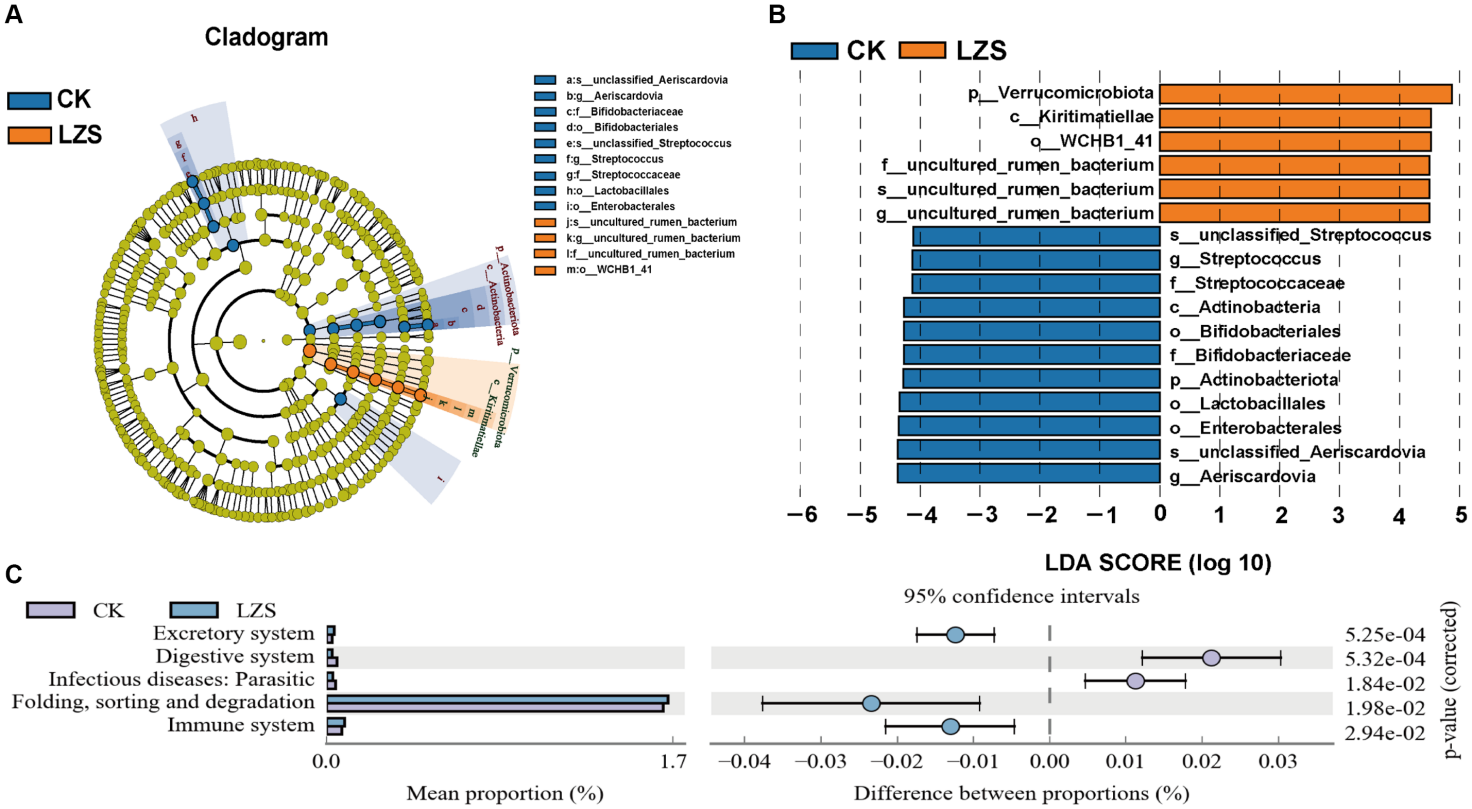
**(A)** Evolution cladogram. **(B)** LDA score chart, LDA > 4, *p* < 0.05. **(C)** Differential analysis of KEGG metabolic pathways graph.

Based on the results of cluster analysis, we performed LEfSe analysis on LZS and CK groups. It was found that the significantly enriched bacteria in the CK group were mainly *Aeriscardovia*, *Streptococcus* and Enterobacterales and were significantly higher than those in the LZS group (LDA > 4, *p* < 0.05; Figures 3A,B). The LZS group Verrucomicrobiota, Kiritimatiellae, *WCHBI_41*, and *uncultured_rumen_bacterium* were significantly higher in abundance than in the CK group (LDA > 4, *p* < 0.05). The LZS and CK groups contained taxa with significant differences in microbial communities for abundance, which may be related to the feeding of PAS. In order to investigate the effect of feeding PAS on intestinal metabolic pathways, we used a *p*-value of < 0.05 as a criterion for differential enrichment analysis. As shown in Figure 3C, Digestive system and Infectious diseases: Parasitic were significantly higher in the CK group than in the LZS group (*p* < 0.05); whereas the LZS group was significantly higher than the CK group (*p* < 0.05) in Excretory system, Folding, sorting and degradation, and Immune system pathways were significantly higher in CK group (*p* < 0.05).

### 3.4. Sequencing results of large and small intestinal segments and bacterial diversity

**Figure 4:**
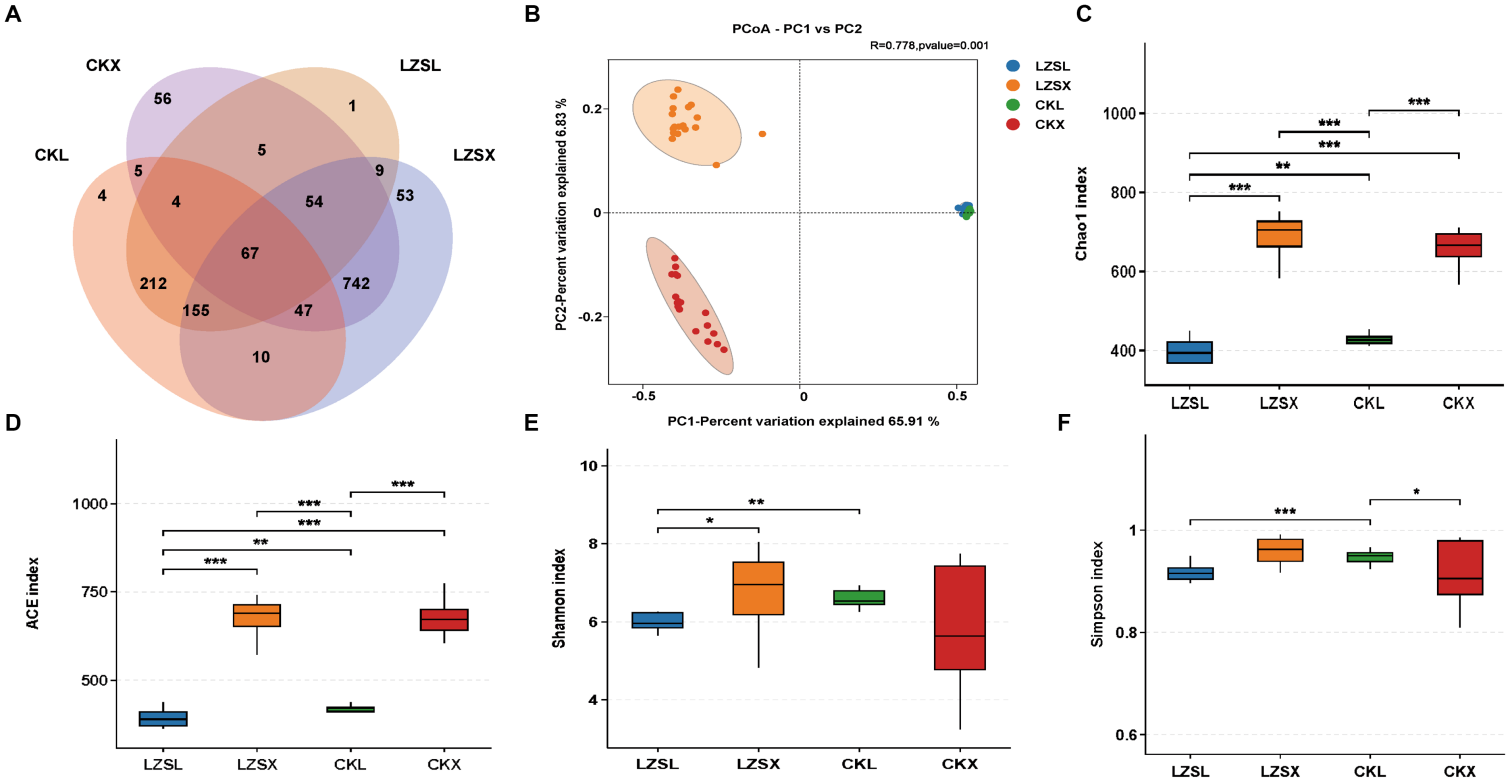
Sequencing results and statistical analysis of diversity. **(A)** Venn diagram showing OTUs shared by CK and LZS groups in large and small intestinal segments. **(B)** Principal coordinate analysis (PCoA) based on all groups. **(C–F)** Chao1, ACE, Shannon index and Simpson index for each group. Adjusted *p < 0.05 and **p < 0.01 by Student’s T-test.

To study the effect of PAS on large and small intestine, respectively. We analyzed 1,424 Features and found that the number of Features observed in the large intestine of the CK group, the small intestine of the CK group, the large intestine of the LZS group, and the small intestine of the LZS group were 504, 980, 507, and 1,137, respectively (Figure 4A). The number of features unique to the small intestine in the CK group and the small intestine in the LZS group were both greater than the large intestine. We then performed a gut microbial diversity analysis (Figures 4C–F). The results showed that the Shannon, ACE, Simpson and Chao1 indices were significantly higher in the CK group than in the LZS group for the large intestinal segment (*p* < 0.05), and significantly higher in the small intestinal segment than in the large intestinal segment for the CK group and the LZS group for the ACE and Chao1 indices (*p* < 0.05). Additionally it was shown by the dilution curve that the sequencing data could be used; Finally, the PCoA analysis revealed that the differences explained by principal component 1 (PC1) and principal component 2 (PC2) were 65.91 and 6.83%, respectively (Figure 4B). The results showed that the small intestine had a higher abundance and diversity of gut microorganisms than the large intestine, and that the flora of the small and large intestines did not correlate well.

### 3.5. Effect of PAS on the abundance of large intestine microbiome

**Figure 5:**
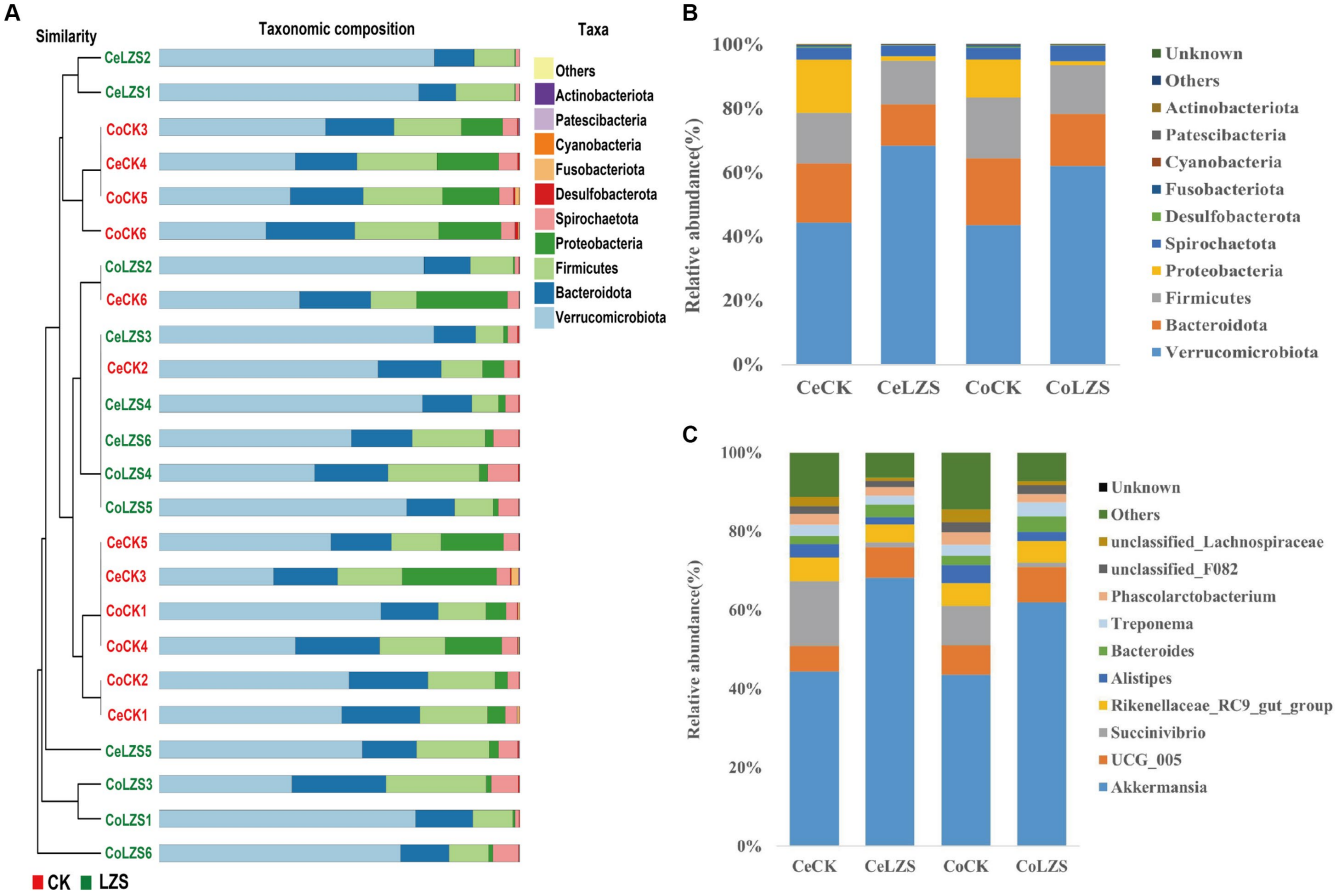
Cluster analysis. **(A)** UPGMA analysis of bacterial communities based on binary_jaccard distance. **(B)** Phylum-level microbial composition of large intestine segments of CK and LZS groups. **(C)** Genus-level microbial composition of the large intestine segments of the CK and LZS groups.

**Figure 6:**
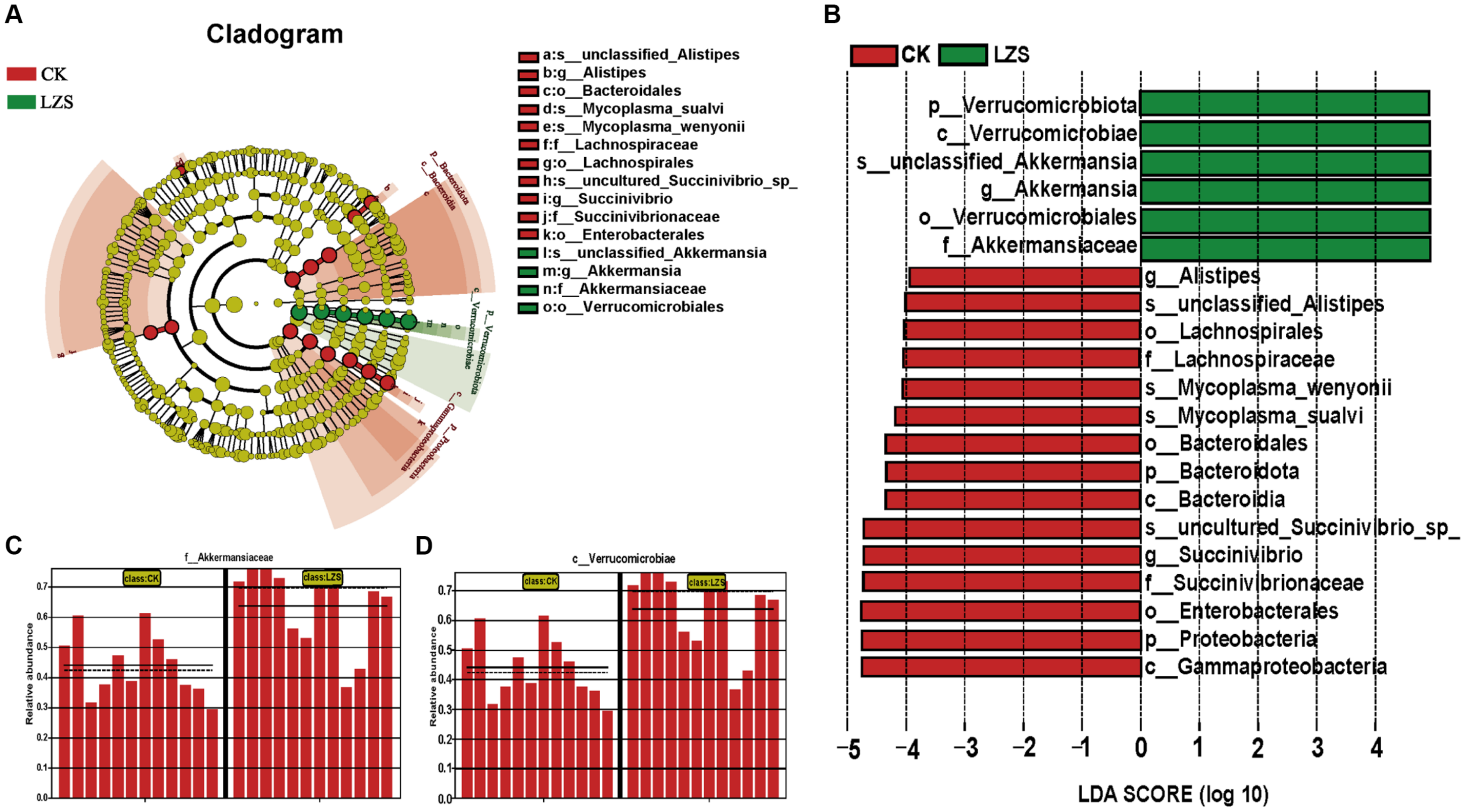
**(A)** Evolution cladogram. **(B)** LDA score chart, LDA > 4, *p* < 0.05. **(C)** Comparison of *Akkermansiaceae* content between CK and LZS groups. **(D)** Comparison of *Verrucomicrobiae* content between CK and LZS groups.

In the large intestine, by performing UPGMA analysis at the phylum level, it was found that PAS had some effect on the important flora of the large intestinal segments, but Verrucomicrobiota was predominant in all samples, followed by Bacteroidota, Firmicutes, Proteobacteria (Figure 5A). Then explore the top 10 bacteria at the phylum and genus level in microbial composition. At the genus level (Figure 5C), the colon and cecum were affected by PAS to essentially the same extent. For example, the addition of PAS resulted in a much higher abundance of both *Akkermansis* as the dominant group, an increase in the abundance of both *UCG_005* and *Bacteroides*, and a decrease in the abundance of both *Succinivibrio* and *Alistipes*. At the phylum level (Figure 5B), the addition of PAS resulted in a significant increase in the abundance of Verrucomicrobiota, a significant decrease in the abundance of Proteobacteria, and little change in the abundance of Bacteroidota and Firmicutes. Finally, based on the results of the cluster analysis, we performed LEfSe analysis on the LZS and CK groups (Figure 6A). The bacteria found to be significantly enriched in the LZS group were *Akkermansiaceae* (Figure 6C) and Verrucomicrobiota (Figure 6D), both belonging to the phylum Verrucomicrobia, Bacteria significantly enriched in the CK group were mainly Gammaproteobacteria, Enterobacteriales and succinivibrionaceae, and all belonged to the phylum Proteobacteria. Therefore, we concluded that the addition of PAS changed the dominant small intestinal flora of sheep from Proteobacteria to Verrucomicrobia (Figure 6B).

### 3.6. Effect of PAS on the abundance of small intestine microbiome

**Figure 7:**
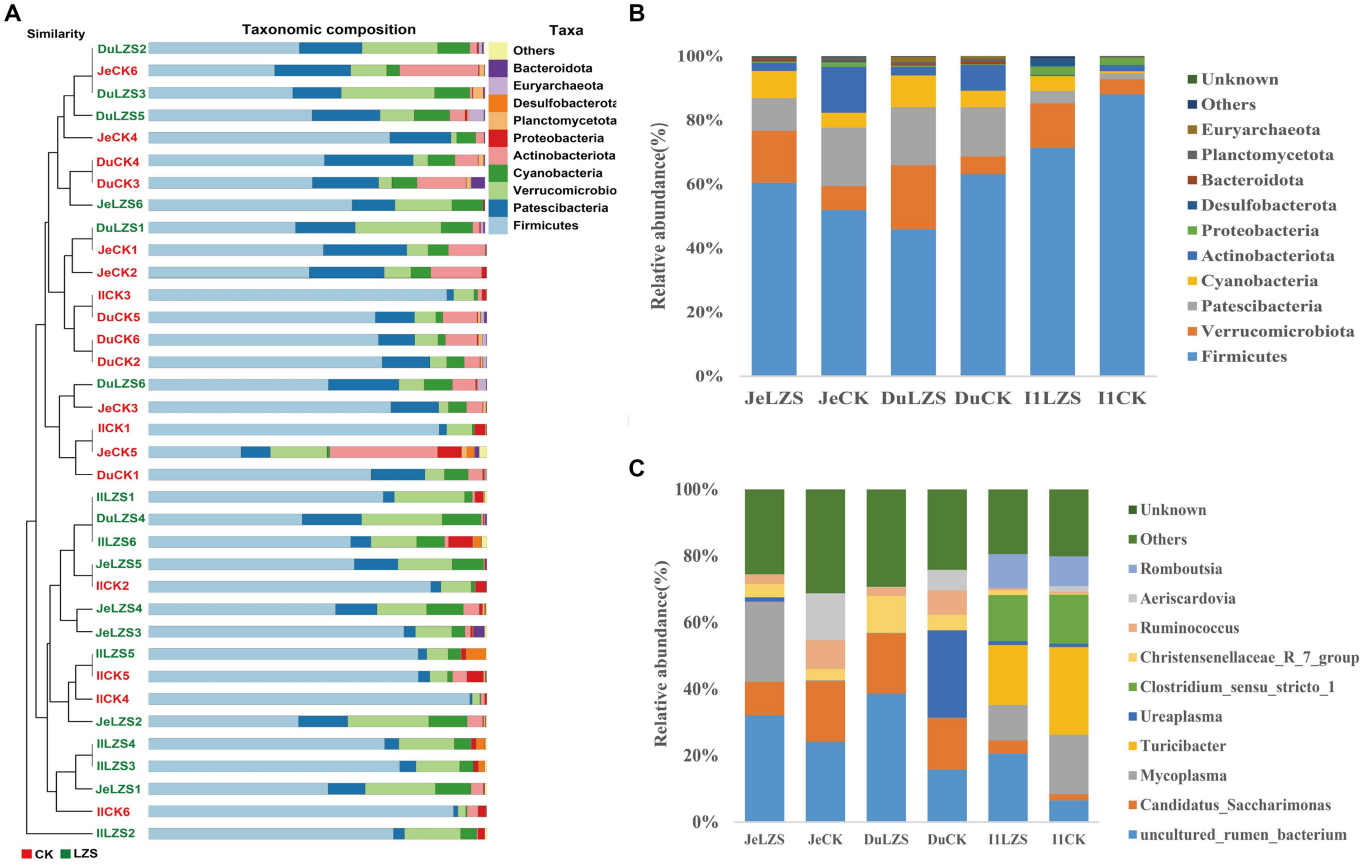
Cluster analysis. **(A)** UPGMA analysis of bacterial communities based on binary_jaccard distance. **(B)** Phylum-level microbial composition of small intestine segments of CK and LZS groups. **(C)** Genus-level microbial composition of the small intestine segments of the CK and LZS groups.

**Figure 8:**
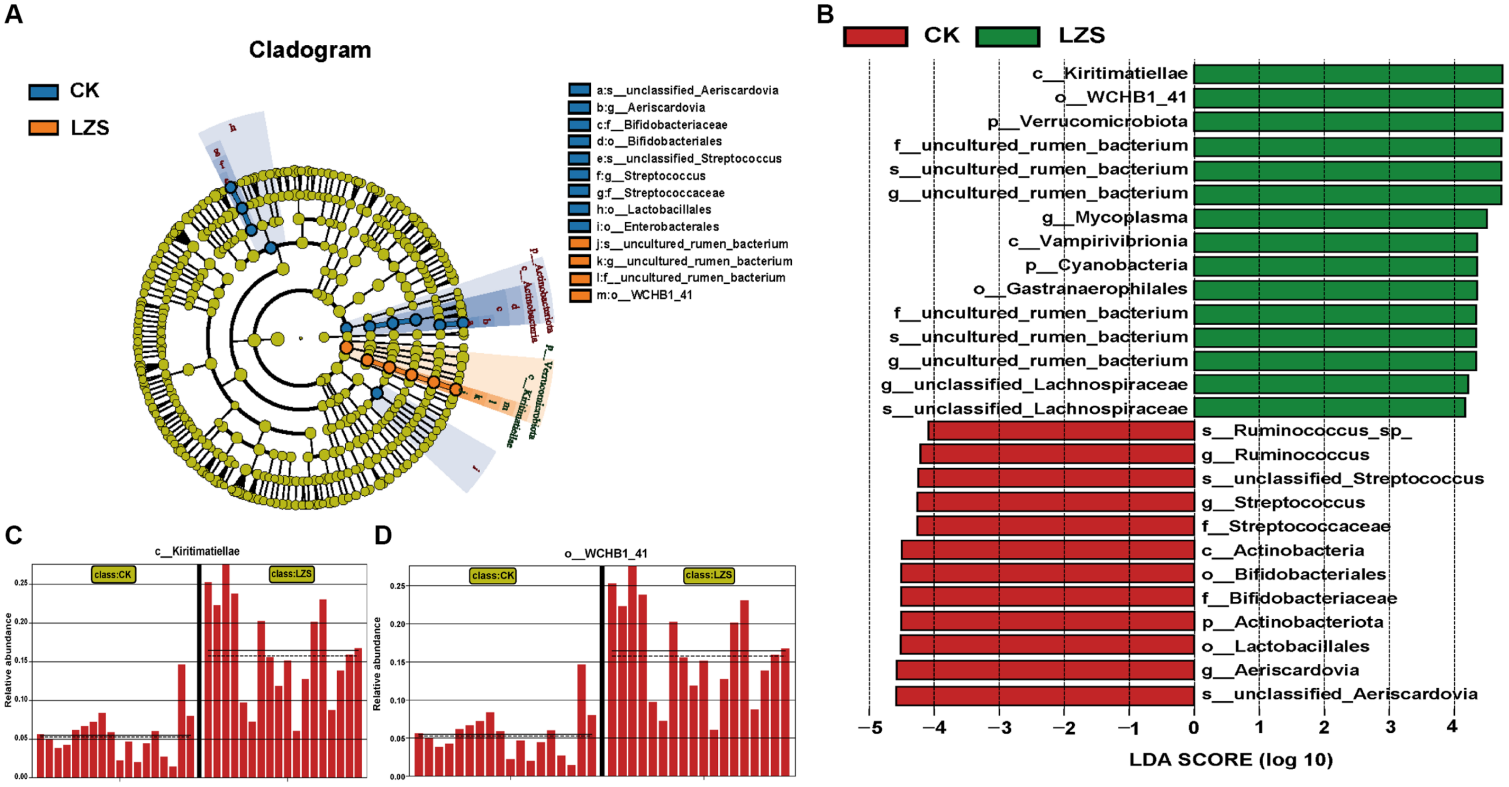
**(A)** Evolution cladogram. **(B)** LDA score chart, LDA > 4, *p* < 0.05. **(C)** Comparison of Kiritimatiellae content between CK and LZS groups. **(D)** Comparison of WCHB1-41 content between CK and LZS groups.

In the small intestine, by performing UPGMA analysis at the phylum level (Figure 7A), it was observed that there was also some variation in the samples from the small intestine segments, but the samples were dominated by Firmicutes, followed by Patescibacteris, Verrucomicrobiota, Cyanobacteria, and others. At the phylum level (Figure 7B), the duodenum, jejunum, and ileum underwent essentially the same changes under the influence of feeding PAS. Firmicutes were the dominant group in both the CK and LZS groups, and their abundance was higher in the CK group than in the LZS group. The abundance of Verrucomicrobiota and Cyanobacteria was increased by the effects of PAS. In addition the abundance of Actinobacteriota showed a decrease under the effect of PAS. At the genus level (Figure 7C), the duodenum, jejunum, and ileum differed considerably in the changes that occurred under the influence of feeding PAS. Among them, *Mycoplasma* declined in the ileum but was significantly elevated in the jejunum. The abundance of *Turicibacter* declined only in the ileum, and the abundance of *Ureaplasma* declined only in the duodenum. Interestingly though the abundance of *Christensenellaceae_R_7_group* was significantly increased in all three intestinal segments. Finally, based on the results of the cluster analysis, we performed LEfSe analysis on the LZS and CK groups (Figure 8A). Bacteria found to be significantly abundant in the LZS group were Kiritimatiellae (Figure 8C), WCHB1_41 (Figure 8D) and Verrucomicrobiota. Bacteria found to be significantly enriched in the LZS group were Kiritimatiellae, WCHB1_41, and Verrucomicrobiota. Bacteria significantly enriched in the CK group were mainly Lactobacillales, *Aeriscardovia*, Actinobacteriota, *Streptococcus*, and Bifidobacteriales (Figure 8B).

### 3.7. Analysis of the correlation between inflammatory factors and gut microbial

**Figure 9:**
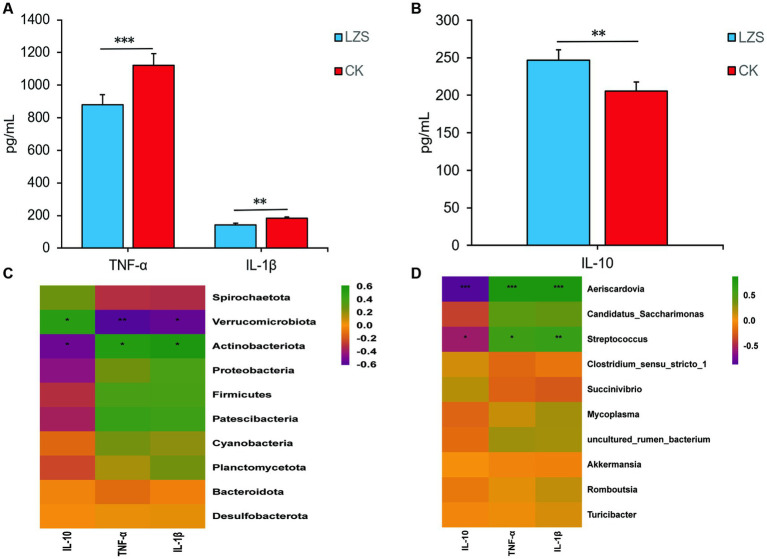
**(A)** Results of changes in pro-inflammatory factor levels in the CK and LZS groups. **(B)** Results of changes in anti-inflammatory factor levels in the CK and LZS groups. **(C)** Heat map analysis results of inflammation factors associated with gut microbiota at the phylum level. **(D)** Heat map analysis results of inflammation factors associated with gut microbiota at the genus level. Adjusted *p value < 0.05 and **p < 0.01 by Student’s T test.

Changes of inflammatory factors (TNF-α, IL-1β, and IL-10) were measured by enzyme-linked immunosorbent assay (Figures 9A,B). The levels of TNF-α and IL-1β in the intestinal tissues of sheep in the LZS group were significantly lower than those of the CK group (*p* < 0.05); The level of IL-10 in the intestinal tissues of sheep was significantly lower than that of the LZS group (*p* < 0.05). Inflammatory factors were correlated with the top 10 gut flora phylum and genus level abundance (Figures 9C,D). The results showed that IL-1β and TNF-α were significantly different (*p* < 0.05) and negatively correlated (*r* < 0) with Verrucomicrobiota. IL-10 was significantly different (*p* < 0.05) and positively correlated (*r* > 0) with Verrucomicrobiota. TNF-α and IL-1β were significantly different (*p* < 0.05) and positively correlated (*r* > 0) with Actinobacteriota, *Aeriscardovia*, and *Streptococcus*. IL-10 was significantly different (*p* < 0.05) and negatively correlated (*r* < 0) with Actinobacteriota, *Aeriscardovia*, and *Streptococcus*.

## 4. Discussion

Increased α-diversity of the gut microbiota is thought to be closely associated with intestinal homeostasis and health. A high diversity of intestinal flora can effectively resist the attack of harmful bacteria, improve intestinal immunity, and promote food digestion and absorption. A high diversity of flora also contributes to the maintenance of gut microbial balance and overall health. In this study, the diversity and richness of gut microorganisms were increased by the addition of PAS. It indicates that PAS helps to improve intestinal immunity and maintain intestinal microbial balance in sheep lambs. The Ace index was lower in the LZS group than in the CK group, probably because ACE considered a wider range of rare species and made corrections for coefficient of variation and sample coverage. Both PCoA and NMDS analysis results showed that PAS altered the community composition of gut microbiota in Hu sheep. Heat map analysis shows significant differences in the microbiota of the small intestine and large intestine. Comparing the species distribution of LZS group and CK group at the phylum level, it was found that Firmicutes were the most important flora in the intestine, which was also found in other mammals (Chang et al., 2020; Minozzi et al., 2020). Firmicutes is an efficient energy harvesting bacterium, which is closely related to the energy acquisition and immune response regulation of the body. The increased abundance of Firmicutes is to meet the energy demand of muscle growth (Du et al., 2022). PAS increased the number of Verrucomicrobiota and Cyanobacteria in the intestine. Verrucomicrobia is a newly classified bacterium, which exists in the intestinal mucosa of healthy people, and has also been found in the Gut microbiota of Tan sheep, blue sheep (*Pseudois nayaur*) and other groups (Wang et al., 2023; Zhao et al., 2023). Research has found that Verrucomimicrobia has anti-inflammatory effects and can maintain glucose homeostasis in the host microbiota (Wang X. et al., 2022). *Christensenellaceae_R_7_group* as highly efficient hydrogen-producing bacteria, and when enriched with methanogens at the same time, they were able to work synergistically to improve the efficiency of enteric fermentation of starch and other polysaccharides (Li et al., 2023). Cyanobacteria have a number of specialized functions, including vitamin B speciali K synthesis, specialized anaerobic fermentation, coprophilic H2 production y and nitrogen fixation. It was also found that cyanobacteria are one of the main gates of gut microorganisms in sheep (Di Rienzi et al., 2013). At the genus level, PAS increased the number of Gut microbiota *Christensenellaceae_R_7_group* and *Romboutsia*. *Romboutsia* was an ileum-specific genus of bacteria. *Romboutsia* has effects on the inflammatory process and is inversely related to inflammatory bowel disease (Schirmer et al., 2019). *Christensenellaceae_R_7_group* is a highly efficient hydrogen producing bacterium. When it is enriched with Methanogen, they can synergistically improve the intestinal fermentation efficiency of starch and other polysaccharides (Li et al., 2023). These results are basically consistent with previous studies, indicating that adding prickly ash products can have beneficial effects on the gut of sheep (Zhang et al., 2022b). LEfSe analysis showed that the LZS group was significantly enriched with bacterial communities such as *Verrucomimicrobiota*, *Kiritimatiellae*, and *WCHBI_41*. Research has shown that Kiritimatellae plays an important role in the digestion process of herbivores (Wang X. et al., 2022), while Akkermansia and Kiritimatellaeota are involved in the biosynthesis pathway of arginine and fatty acids (Baniel et al., 2021). WCHB1-41 is significantly affected by lifestyle changes (Zhang C. et al., 2023), Akkermannia and WCHB1-41 encoded arginine and Fatty acid synthesis metabolic pathway can effectively improve energy and nitrogen utilization efficiency of yaks (Guo et al., 2021). By analyzing the differences in KEGG metabolic pathways, it was found that the proportions of metabolic pathways of Excretory system, Folding, sorting and degradation, and Immune system in the LZS group were significantly higher than those in the CK group (*p* < 0.05), and that the proportions of metabolic pathways of Digestive system and Infectious diseases:parasitic were significantly higher than those in the CK group (*p* < 0.05). The proportion of metabolic pathways in Digestive system and Infectious diseases:parasitic was significantly higher than that in LZS group (*p* < 0.05). It shows that PAS can indeed improve the immunity of Hu sheep lambs, reduce the Parasitic disease of Hu sheep lambs (He et al., 2018; Patil and Mallya, 2022), and at the same time, PAS also has a certain impact on the digestion and excretion of sheep. However, PICRUSt2 still has shortcomings in predicting potential functions. Therefore, it is necessary to further study the impact of PAS on the metabolic ability of Hu sheep lambs.

After performing ELISA analysis of inflammatory factors, the results showed a significant increase in IL-10 levels (*p* < 0.05) and a significant decrease in IL-1β and TNF-α levels (*p* < 0.05) in the LZS group compared to the CK group. IL-10 prevents colitis by eliminating dysfunctional mitochondria and inhibiting mTOR signaling and inflammatory vesicle activation in macrophages (Ip et al., 2017). Elevated levels of IL-10 indicated an improvement in the immune mechanism of Hu sheep by PAS. In the context of chronic inflammation, high circulating expression of IL-1β promotes the production of oncogenic mediators to induce tumorigenesis, but IL-1β can also promote T cell-mediated adaptive immune responses (Kallapur et al., 2011). TNF-α is closely associated with intestinal flora disorders. It can modulate immunity by promoting IL-10 production by B cells, inducing T cell apoptosis, altering T cell receptor (TCR) signaling, inhibiting T helper 17 (Th17) cell differentiation, and enhancing the number and function of regulatory T (Treg) cells (Galinsky et al., 2020). Elevated levels of IL-1β and TNF-α similarly indicated a positive effect of PAS on the immunocompetence of Hu sheep (Zhang et al., 2021). After conducting correlation heat map analysis, we found very interesting phenomena. Verrucomimicrobiota, as a differential phylum of gut microbiota, has certain anti-inflammatory effects, IL-10 is a recognized inflammatory and immunosuppressive factor (Zheng et al., 2019). We intuitively found them to be positively correlated through heat maps, which has also been confirmed; IL-1β and TNF-α belong to the TNF ligand superfamily and are both pleiotropic pro-inflammatory cytokines (Barichello et al., 2010). Streptococcuis an important pathogen that causes bacterial meningitis. *Actinobacteriota* and *Aeriscardovia* may also have certain pro-inflammatory effects (Liu et al., 2023). It was inferred that there may be some connection between inflammatory factors and intestinal flora composition and that PAS has a positive effect on resistance to inflammatory bowel disease in Hu sheep.

Because of the differences in the action functions of the small and large intestine (Yang et al., 2021), after exploring the effects of PAS on intestinal microorganisms, we focused our analysis on individual intestinal segments. It was found that the number of features unique to the small intestine segments of the CK group and the small intestine segments of the LZS group were both greater than that of the large intestine segments, and that the small intestine had a higher abundance and diversity of intestinal microorganisms than the large intestine. PCoA showed that the gut microbial population structure in the large intestine segments of the test and control groups was essentially the same. For the large intestinal segments, the microbial communities of the cecum and colon changed almost identically after feeding PAS. This is basically the same as other scholarly studies (Ma et al., 2022). At the phylum and genus level, the predominant colony in the cecum and colon was Verrucomicrobiota, and this colony increased considerably in both the cecum and colon with the addition of PAS. Proteobacteria are positively correlated with fiber intake (Zhao et al., 2022); *Akkermansia*, in the cecum and colon, was also significantly increased by PAS, which promotes intestinal barrier integrity, modulates immune responses, suppresses inflammation, and supports an anti-inflammatory gut microbiota (Xi et al., 2020; Hagi and Belzer, 2021; Kalia et al., 2022). There was also an increase in UCG-005, a member of the Ruminococcaceae family, which is associated with cellulose and starch digestion (Sun et al., 2019; Liu and Yin, 2021). LEfSe analysis showed a shift in the dominant flora of the large intestine segment from *Bacteroidetes* and *Gammaproteobacteria* to *Akkermansiaceae* and *Verrucomicrobiae*. Bacteroidetes live in the intestines of animals, and studies have found that Bacteroidetes promote digestion and increased utilization of complex carbohydrates, and are associated with obesity levels (Hales et al., 2017; Zhang et al., 2018). *Gammaproteobacteria*, belonging to Pseudomonadota in the bacterial kingdom, usually exists in the digestive tract and can metabolize cancer drugs, drive immunosuppression and tumorigenesis. *Gammaproteobacteria* can induce cell damage by infecting pancreatic cells, even hiding within pancreatic cells, causing double stranded DNA breakage (Halimi et al., 2021). Interestingly, the microbial communities in the duodenum, jejunum and ileum of the small intestine were changed after feeding PAS. We then found that at the phylum level, the dominant flora in small intestine is *Firmicutes*, which has been demonstrated in multiple studies to be the dominant intestinal phylum for the breakdown of complex nutrients and the production of beneficial metabolites (Gebeyew et al., 2021); At the genus level, the dominant genus in the small intestine is *uncultured_rumen_bacterium*, which is in general agreement with other scholarly studies (Kumar et al., 2022). Also *ureaplasma* is a harmful bacterium that can cause inflammation of the fetal membranes (Snyder et al., 2013; Heiter et al., 2023). LEfSe analysis result showed that *Kiritimatiellae* and *WCHB1_41* significantly enriched in the LZS group while *Lactobacillales* and *Aeriscardovia* significantly enriched in the CK group. Lactobacillales belongs to the class Bacilli and can improve sheep manure composting efficiency and final product quality and can influence mitochondrial biogenesis to improve meat quality in sheep (Li et al., 2020, Wang C. et al., 2022). *Aeriscardovia* belongs to the family Bifidobacteriaceae and is found in omnivorous organisms that consume a wide range of nutrients and are capable of expanding their metabolism of different carbon sources present in the host (Lugli et al., 2017). In summary, adding PAS to the diets can increase beneficial flora content such as Verrucomicrobia, Firmicutes, Kiritimatiellae, *Christensenellaceae_R_7_group*, *WCHBI_41*, *UCG-005* and *Akkermansia*, which in turn improves the sheep intestinal tract, and provides a new view on the improvement of sheep immunity.

## 5. Conclusion

This study revealed the effect of PAS addition on intestinal bacteria in Hu sheep lambs and validated the associated inflammatory factors. It was found that the addition of PAS could increase the diversity and abundance of intestinal microorganisms, influence the composition of intestinal community in sheep, and increase the content of beneficial intestinal bacteria. Moreover, the excretory system and immune system were significantly enhanced. The ELISA test showed that PAS has a certain impact on the levels of inflammatory factors. Then we explored the effects of PAS on the small and large intestines, respectively, and found that for both CK and LZS groups, they showed that the number of unique features of the small intestinal segment was more than that of the large intestinal segment, and that the gut microbial abundance and diversity in the small intestines were higher than that in the large intestines. Moreover, the addition of PAS significantly increased *Akkermansiaceae* and *Verrucomicrobiae* in the large intestine segment, and Kiritimatiellae and *WCHB1 41* in the small intestine segment. Thus, it can be confirmed that feeding PAS promotes changes in the abundance of intestinal microorganisms in sheep, affects the levels of inflammatory factors and thus improves immunity in sheep lambs. This study provides new insights into improving the stress resistance and intestinal health of sheep by adding green additives, and provides a theoretical basis for understanding how ruminant gut microbiota are affected by PAS.

## Data availability statement

The datasets presented in this study can be found in online repositories. The names of the repository/repositories and accession number(s) can be found below: NCBI - PRJNA1003863.

## Ethics statement

The animal studies were approved by Animal Care Committee of Gansu Agricultural University (GSAU-AEW-2020-0057). The studies were conducted in accordance with the local legislation and institutional requirements. Written informed consent was obtained from the owners for the participation of their animals in this study.

## Author contributions

DL: Methodology, Writing – original draft, Writing – review & editing. HY: Writing – review & editing. QL: Writing – review & editing. KM: Writing – review & editing. HW: Writing – review & editing. CW: Writing – review & editing. TL: Writing – original draft, Writing – review & editing. YM: Methodology, Writing – original draft, Writing – review & editing.

## Supplementary material

Supplementary figure 1 Rarefaction curves of all samples. (*n* = 60).

Supplementary FIGURE 2 Histogram of genus level Species distribution of all samples. (*n* = 60).
