# Supplementary figures and images for: Prickly Ash Seeds improve immunity of Hu sheep by changing the diversity and structure of gut microbiota
Source: Front Microbiol. 2023 Oct 31;14:1273714. doi: 10.3389/fmicb.2023.1273714 (PMC10644117; doi:10.3389/fmicb.2023.1273714)

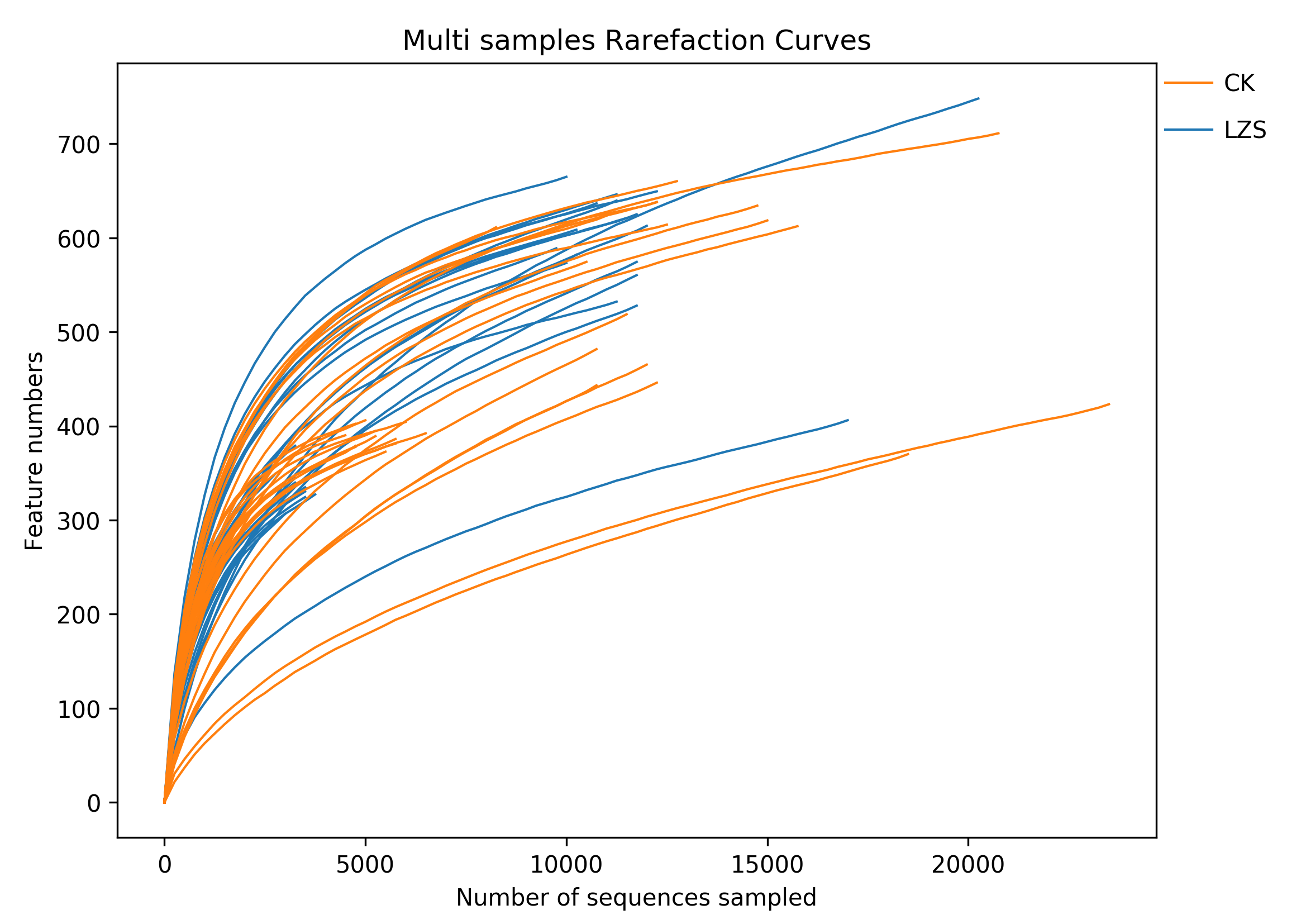

Supplement: Supplementary figure 1 — Rarefaction curves of all samples. (n = 60). [file Image_1.PNG]

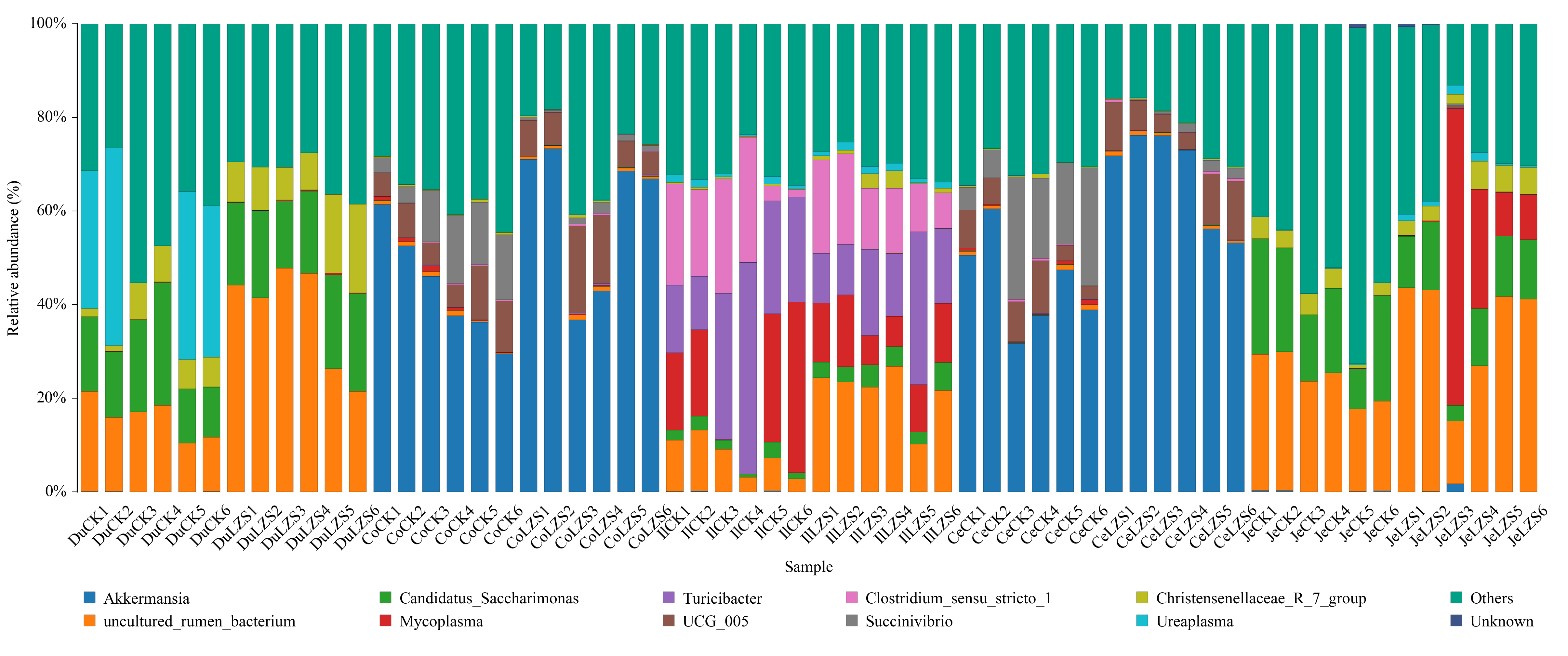

Supplement: Supplementary FIGURE 2 — Histogram of genus level Species distribution of all samples. (n = 60). [file Image_2.PNG]
